# Systems genetics analysis reveals the common genetic basis for pain sensitivity and cognitive function

**DOI:** 10.1111/cns.14557

**Published:** 2024-02-07

**Authors:** Fuyi Xu, Anran Chen, Shuijing Pan, Yingying Wu, Hongjie He, Zhe Han, Lu Lu, Buyan‐Ochir Orgil, XiaoDong Chi, Cunhua Yang, Shushan Jia, Cuicui Yu, Jia Mi

**Affiliations:** ^1^ Shandong Technology Innovation Center of Molecular Targeting and Intelligent Diagnosis and Treatment Binzhou Medical University Yantai China; ^2^ The Affiliated Yantai Yuhuangding Hospital of Qingdao University Yantai China; ^3^ University of Tennessee Health Science Center Memphis Tennessee USA; ^4^ Department of Anesthesiology YanTai Affiliated Hospital of BinZhou Medical University Yantai China

**Keywords:** BXD mice, cognition, hippocampus, pain sensitivity, *Rnf20*

## Abstract

**Background:**

There is growing evidence of a strong correlation between pain sensitivity and cognitive function under both physiological and pathological conditions. However, the detailed mechanisms remain largely unknown. In the current study, we sought to explore candidate genes and common molecular mechanisms underlying pain sensitivity and cognitive function with a transcriptome‐wide association study using recombinant inbred mice from the BXD family.

**Methods:**

The pain sensitivity determined by Hargreaves' paw withdrawal test and cognition‐related phenotypes were systematically analyzed in 60 strains of BXD mice and correlated with hippocampus transcriptomes, followed by quantitative trait locus (QTL) mapping and systems genetics analysis.

**Results:**

The pain sensitivity showed significant variability across the BXD strains and co‐varies with cognitive traits. Pain sensitivity correlated hippocampual genes showed a significant involvement in cognition‐related pathways, including glutamatergic synapse, and PI3K‐Akt signaling pathway. Moreover, QTL mapping identified a genomic region on chromosome 4, potentially regulating the variation of pain sensitivity. Integrative analysis of expression QTL mapping, correlation analysis, and Bayesian network modeling identified Ring finger protein 20 (Rnf20) as the best candidate. Further pathway analysis indicated that *Rnf20* may regulate the expression of pain sensitivity and cognitive function through the PI3K‐Akt signaling pathway, particularly through interactions with genes *Ppp2r2b*, *Ppp2r5c*, *Col9a3*, *Met*, *Rps6*, *Tnc*, and *Kras.*

**Conclusions:**

Our study demonstrated that pain sensitivity is associated with genetic background and *Rnf20‐*mediated PI3K‐Akt signaling may involve in the regulation of pain sensitivity and cognitive functions.

## Graphical abstract

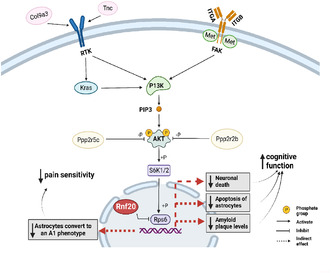

## 1 INTRODUCTION

The sensation of pain begins with the transduction of a stimulus in the periphery nerves and is converted to electrical signals that travel to the central nervous system. In the brain, the sensory signals can be encoded and combined in the major brain compartments that involve in learning and memory to form pain‐related memory.^1^ Thus, pain is not only an unpleasant sensory but also a subjective perceptive phenomenon involving cognitive processing.^2^, ^3^ On the other hand, cognitive impairment also influences pain experience in patients with acute pain or with chronic pain syndrome,^4^ and is also associated with the individual variation of pain sensitivity.^4^, ^5^ Several studies have shown an opposite relationship between pain and cognitive performance and significant negative effects of pain on cognition in healthy subjects have been recognized.^6^, ^7^ Such associations may be explained by the potential common mechanism(s) that drive pain sensation and cognitive processing in the brain.

As the major brain compartment involving learning and memory, the hippocampus is closely associated with the experience of pain.^8^, ^9^ The activation of the hippocampus is related to individual sensitivity to pain expectancy and assessment of the stimulus value.^9^ Moreover, individuals who are highly sensitive to pain expectations show more active and extensive hippocampal activity.^9^ Animal studies further confirmed the hippocampal influence on neuropathic pain behaviors.^10^ For instance, dysfunction of plectin isoform P1c in hippocampal neurons can lead to decreased thermal pain sensitivity and impaired cognitive functions.^11^ However, the association between hippocampal gene expression and individual pain perception variance remains largely unknown.

Transcriptome‐wide association study (TWAS) is an emerging approach for creating phenotype‐gene correlation networks to reveal molecular mechanisms and causal genes of diseases at a population level.^12^ However, due to the difficulty in accessing human tissues for transcriptome assays, mouse genetic reference populations are more widely used. Among which, the BXD panel of recombinant inbred (RI) mice together with their parental strains, C57BL/6J (B6) and DBA/2J (D2), provide a unique platform with more than 150 stains are available and thousands of transcriptome/phenome data sets that have been published over the past decades, including 250 phenotypes related to cognitive functions.^13^ In addition, as each BXD strain has been stably inbred and can be replicated in large numbers as desired, facilitating the precise mapping of complex traits with low to moderate heritability.^14^ Moreover, the mice population of this panel presents strong variations in pain sensitivity, indicating it is a suitable population for revealing the potential mechanisms that underlie the association between pain sensitivity and cognitive function.

This study aimed to identify common functional genes involved in pain sensitivity and cognitive functions and uncover shared molecular mechanisms that underlie those phenotypes. By employing the TWAS, QTL/eQTL mapping, and Bayesian network modeling in the BXD RI mice, we ultimately identified *Rnf20* as a genetic regulator that modulates the pain sensitivity and cognitive function through the PI3K‐Akt signaling pathway.

## 2 MATERIALS AND METHODS

### 2.1 Measurement of pain sensitivity

A total of 60 BXD RI strains were used to evaluate the variation of pain sensitivity. Three to ten male mice from each strain were selected to participate in Hargreaves' paw withdrawal test at 8–9 weeks of age. The paw retract latency was used as an indicator to assess the sensitivity to acute thermal pain,^13^ where the shorter the incubation period for claws to retract, the lower the pain threshold, or greater pain sensitivity.^15^ Briefly, the lights in the testing area were turned off at least one hour prior to testing and animals were allowed to sit undisturbed in the darkened room. A lamp (15 W bulb) behind the hot plate faced away from the hot plate surface. The hot plate was maintained at 52°C. The mouse was placed on the center of the hot plate in a bottomless cube and the built‐in timer started. As soon as the animal showed a pain response (i.e., paw licking, guarding, shaking, or jumping), the timer was immediately stopped and the animal was removed from the hot plate surface. If the animal did not respond within 30 s, the test was stopped, and the animal was assigned the 30 s maximum time as its response latency. Once an animal had been tested, it was placed in a holding cage until all animals from the home cage had been tested.

### 2.2 Quantification of hippocampus transcriptome

The data of the hippocampus transcriptome was obtained from 67 BXD RI strains plus B6 and D2 parents and two reciprocal F1 hybrids. The vast majority of the animals ranged in age from 45 to 90 days (the average was 66 days and the maximum ranged from 41 to 196 days). The mice were given regular health checks and sacrificed by cervical dislocation and brains were removed and placed in RNAlater. Then, the entire hippocampus was dissected for downstream analysis.

RNA was isolated by the RNA STAT‐60 method with the following steps: (1) Homogenize tissue samples in the 0.7 mL RNA STAT‐60; (2) Store the homogenate for 5 min at room temperature. Next, add 0.14 mL of chloroform, cover the sample tightly, shake vigorously for 15 s, and stay at room temperature for 2–3 min. Centrifuge the homogenate at 12,000 *g* for 15 min at 4°C. (3) Transfer the aqueous phase to a fresh tube and mix with 0.35 mL isopropanol. Store samples at room temperature for 5–10 min and centrifuge at 12,000 *g* for 10 min at 4°C; (4) Remove supernatant and wash the RNA pellet once with 0.7 mL 75% ethanol by vertexing and subsequent centrifugation at 7500 *g* for 5 min at 4°C. Dry the RNA pellet briefly in a vacuum and dissolve in water. RNA purity and integrity were evaluated by the 260/280 nm absorbance ratio and Agilent Bioanalyzer 2100. Samples with 260/280 >1.8 and RNA integrity number (RIN) >8 were used for transcriptome profiling on the Affymetrix Mouse Genome 430 2.0 array.

### 2.3 Data normalization

The raw microarray data was processed with the RMA method^16^ to correct the background, normalize, and summarize the results. The expression values were further rescaled with a modified *Z* score method as previous description.^17^ In short, RMAs were transformed into log2‐values. Then the data of each single array was converted to *Z*‐scores, multiplied by 2, and a value of 8 was added.

### 2.4 Correlation analysis

The correlation analysis was performed with the Pearson correlation coefficient to identify trait–trait, gene‐trait, as well as gene–gene associations. For trait–trait correlation analysis, we correlated pain sensitivity to all BXD published phenotypes in GeneNetwork (www.genenetwork.org). For gene‐trait and gene–gene correlation analysis, the pain sensitivity or gene of interest was correlated to the hippocampal transcriptome. The resulting *p*‐value <0.05 was considered statistically significant.

### 2.5 Gene functional enrichment analysis

The functional analysis of the genes of interest was performed using WebGestalt (http://www.webgestalt.org).^18^ The KEGG pathway and Mammalian Phenotype Ontology (MPO) analysis were used to illustrate the pathways or biological phenotypes involved in genes, respectively. Annotations with *p*‐value <0.05 and a minimum overlap of 5 genes were considered significant.

### 2.6 Quantitative trait locus (QTL) and expression QTL (eQTL) mapping

The QTL and eQTL mapping were performed on GeneNetwrok, as described in our previous work.^19^, ^20^, ^21^ The conventional interval mapping method,^22^ which yields a likelihood ratio statistic (LRS) score, was used to measure the confidence of linkage between the observed phenotype and a genomic region. The genome‐wide suggestive and significance thresholds were determined by 1000 permutation tests.

### 2.7 Phenome‐wide association analysis (PheWAS)

PheWAS is a reverse genetic analysis method to identify the potential phenotypes associated with genetic variations.^23^ Genes that contain high‐impact variants, including missense, nonsense, splice site, frameshift mutations, and copy number variations, as well as genes that have significant cis‐e(p)QTLs in the BXD transcriptome datasets were included in the PheWAS analysis. Genetic variants of each gene are represented by the SNPs within the genes as well as their cis‐QTLs. About 5000 clinical phenotypes were used to study the association between genes and phenotypes. In this study, we used a multi‐locus mixed‐model approach (mlmm) to estimate the associations between each gene and clinical traits. This analysis was performed on Systems Genetics at EPFL (https://systems‐genetics.org).

### 2.8 Expression‐based PheWAS (ePhewas)

Associations between transcripts and phenotypic traits were estimated using mixed model regression analysis. Transcript–trait pairs with fewer than 15 overlapping lines were removed from the analysis. Phenotype‐wide significance analysis was performed using Bonferroni correction. This analysis was performed on Systems Genetics at EPFL.

### 2.9 Causative analysis with Bayesian network modeling

The Bayesian network analysis was used to identify the causality among QTL genotype, gene expression, and phenotype. This analysis was done at the Bayesian Network Webserver (BNW; http://compbio.uthsc.edu/BNW/)^24^, ^25^ with the following 10 variables: chromosome 4 QTL peak position genotype (rs27789011), 8 gene expression traits (*Rnf20, Rad23b, Frrs1l, Hdhd3, Alad, Epb41l4b, Atp6v1g1*, and *Pole3*), and one phenotype (pain sensitivity, BXD_11307). The network model structure was learned from the data with the following settings: Each variable had a maximum of 4 direct parents in any potential model, model averaging of the 1000 highest scoring networks was performed, and directed edges with weights >0.5 were included in the final network model.

## 3 RESULTS

### 3.1 Pain sensitivity varied greatly and associated with cognition‐related traits in BXD mice

**FIGURE 1:**
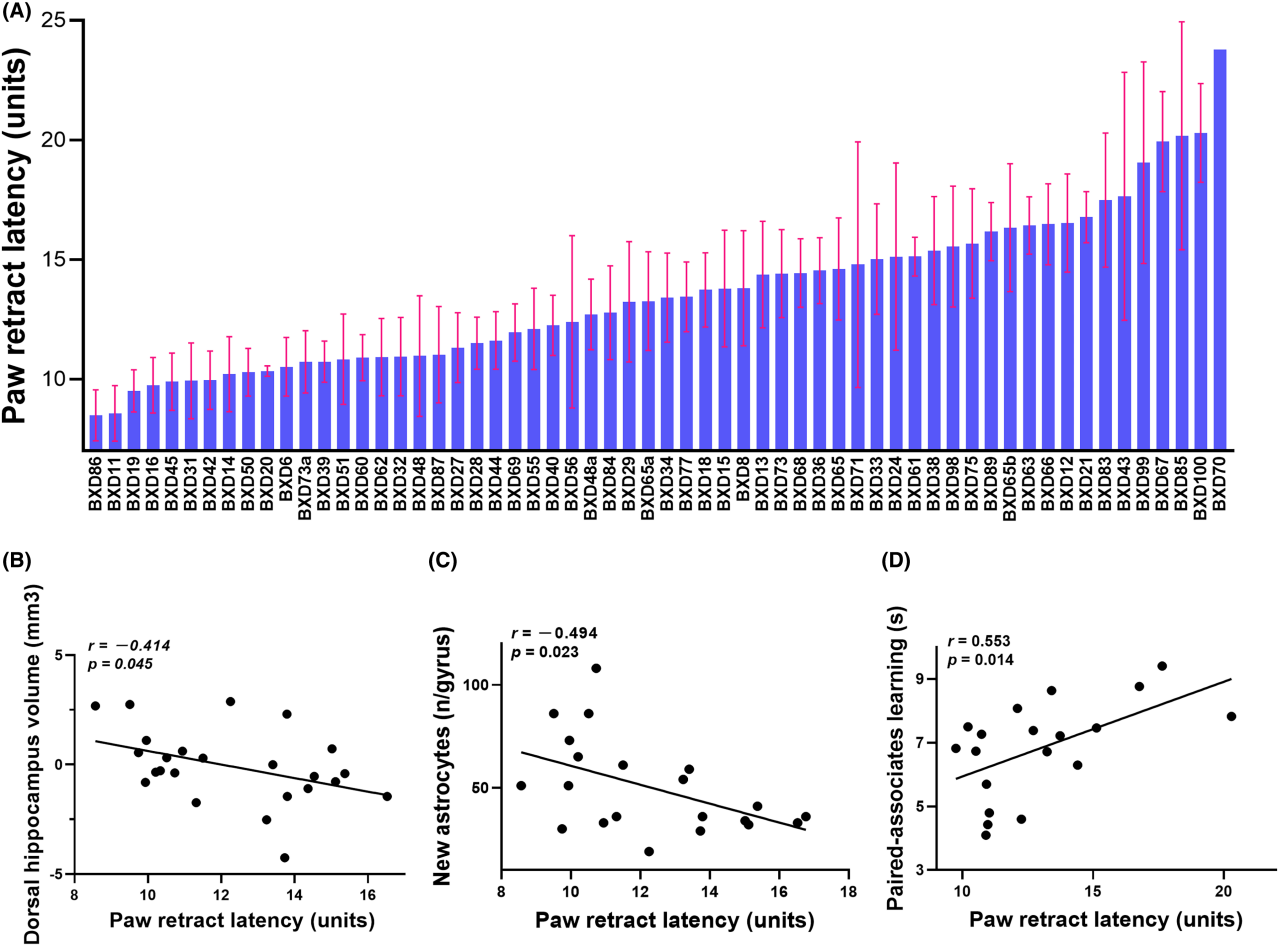
Hargreaves' paw withdrawal test and association of pain sensitivity with cognition‐related traits in BXD mice. (A) Barchart showing the distribution of paw retract latency (*Y*‐axis, s), an indicator of sensitivity to acute thermal pain, across the 60 BXD RI strains (*X*‐axis). Data are shown as mean ± SEM. Three to ten male mice from each strain were participated in Hargreaves' paw withdrawal test at 8–9 weeks of age. The detailed sample size for each strain can be reviewed on GeneNetwork (www.genenetwork.org) with phenotype ID of BXD_11307. (B–D) Scatter plots showing associations between pain sensitivity (*X*‐axis, s) and dorsal hippocampus volume (B, *Y*‐axis, mm^3^), new astrocytes in the dentate gyrus (C, *Y*‐axis, neurons (n) per gyrus), as well as object‐location paired‐associates learning using a touchscreen (D, *Y*‐axis, s). Each dot represents the mean value from 3 to 10 mice/strain. Pearson correlation analysis was used to determine the relationship. Pearson correlation *r* and *p* values are indicated.

Consistent with the inflammatory pain^26^ or mechanosensation^27^ observed in the BXD mice, the Hargreaves' paw withdrawal test revealed significant variations in thermal pain sensitivity among the 60 BXD RI strains studied here (Figure 1A). The mean time to withdrawal of all strains was 13.563 seconds (s) and the median was 13.334 s. BXD86 showed the lowest pain threshold with the time to withdrawal of 8.483 s, whereas BXD70 had the longest time to withdrawal of 23.778 s, showing the lowest pain sensitivity. The overall difference reached 15.295 s.

Pearson correlation analysis revealed that pain sensitivity was significantly associated with the central nervous system‐related phenotypes in the BXD mice such as dorsal hippocampus volume, new astrocytes in the dentate gyrus, and object‐location paired‐associates learning (Figure 1B–D). The dorsal hippocampus volume (*r* = −0.414, *p* = 0.045) and new astrocytes in the dentate gyrus (*r* = −0.494, *p* = 0.023) were negatively correlated with the claw retraction time, indicating that BXD mice with lower pain threshold have larger dorsal hippocampal volume and higher number of new astrocytes in the dentate gyrus. Meanwhile, object‐location paired‐associates learning using a touchscreen assays (*r* = 0.553, *p* = 0.014) showed a significant positive correlation, implying that the more sensitive the BXD RI strains were to pain, the better their learning and memory abilities were.

### 3.2 TWAS indicates pain sensitivity associated genes in the hippocampus involved in cognition‐related functions and pathways

**FIGURE 2:**
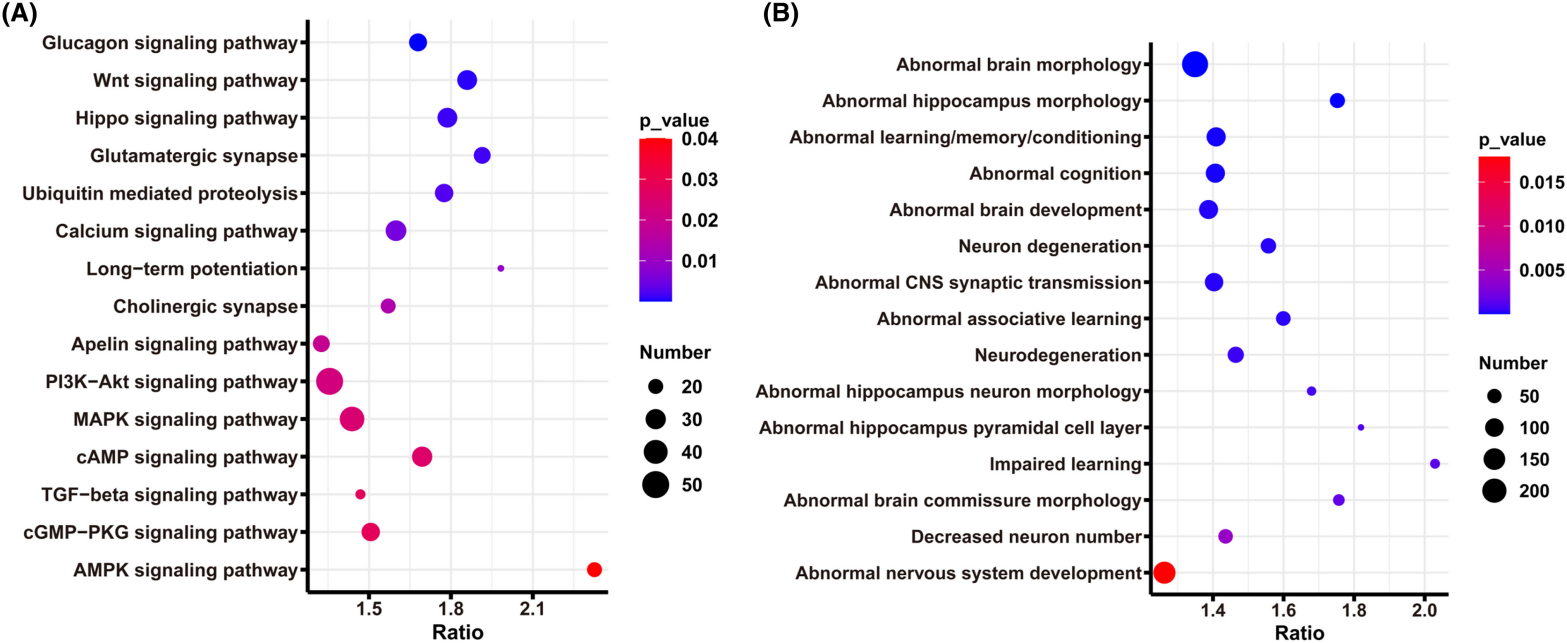
Gene set enrichment analysis of pain sensitivity associated hippocampal genes. The bubble plot shows the enriched KEGG pathways (A) and Mammalian Phenotype Ontology terms (B). The *X*‐axis represents the enrichment ratio, and the *Y*‐axis shows the enriched terms. The size of the dots represents the number of genes, and the color of the dots represents the *p*‐value.

To further explore the associations between pain sensitivity and hippocampal expression genes, we compared values of paw retract latency in BXDs with all 45,101 probes for the Pearson correlation coefficient. This analysis defined 3404 probes (corresponding to 2973 transcripts) as being associated with pain sensitivity (*p* < 0.05). KEGG pathway enrichment analysis showed a significant involvement of those transcripts in cognition‐related pathways (Figure 2A), such as glutamatergic synapse, PI3K‐Akt signaling, MAPK signaling, cAMP signaling, and cGMP‐PKG signaling pathways. In addition, neural or brain structure and function‐related MPO terms such as abnormal brain morphology, abnormal hippocampus morphology, abnormal learning/memory/conditioning, abnormal cognition, abnormal brain development, and neuron degeneration were also enriched (Figure 2B).

Among the top 50 most correlated genes (Table S1), *Tfdp2* had the highest correlation coefficient (*r* = 0.597, *p* = 2.31E‐06). *Tfdp2* is a cofactor required for cell cycle control and its expression has been associated with neurological diseases including Alzheimer's disease (AD).^28^ In addition, *Lats2*, *Fbln1*, *Ptprd*, *Rnf20*, *Coasy*, *Cox10*, *Slitrk1*, *Sfrp1*, *Pdpn*, *Rad23b*, and *N28178* (*Phf24*) have been also implicated in the cognitive function.^29^, ^30^, ^31^, ^32^, ^33^, ^34^, ^35^, ^36^, ^37^, ^38^, ^39^, ^40^ Further, *Ptprd, Akap12, Cadm1, Myo5a, Slitrk1*, and *Pdpn* were involved in “synaptic” related biological processes, with four of them (*Ptprd*, *Akap12*, *Cadm1*, and *Myo5a*) related to the Schaffer collateral‐CA1 synapse, one of the areas with early dysfunction in AD.^41^

### 3.3 A genomic locus on chromosome 4 co‐regulates both pain sensitivity and its associated gene expressions

**FIGURE 3:**
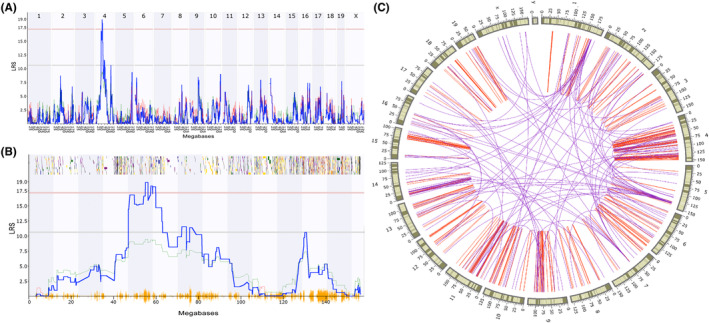
QTL /eQTL mapping of pain sensitivity and its associated gene expressions in BXD strains. (A) Manhattan plot showing a pain sensitivity regulating locus on chromosome 4. (B) Zoomed in the identified QTL on chromosome 4. The *X*‐axis denotes a position on the mouse genome in megabases and the *Y*‐axis indicates the LRS value. The red and gray horizontal lines indicate significant and suggestive thresholds. (C) Circos plot showing genetic regulating loci for pain sensitivity associated genes in the hippocampus. Red represents *cis*‐eQTL and blue represents *trans*‐eQTL. QTL and eQTL mapping were done using GeneNetwork.

To identify the genomic loci controlling the variation of the pain sensitivity across the BXD RI strains, we conducted a genome‐wide composite interval mapping analysis. With the genome‐wide significant threshold of 17.37 determined by 1000 permutation tests, a significant QTL was mapped to chromosome 4 with a peak LRS of 18.92 at the position of 54.951megabases (Mb) (Figure 3A). This QTL encompasses 20 Mb from 46.8 to 65.9 Mb with 1.5 LOD dropoff (Figure 3B).

We also performed eQTL mapping for those ~3000 transcripts associated with pain sensitivity and determined 479 transcripts that showed genetic regulation (LRS > 20, Figure 3C), with 352 being *cis*‐regulated and 127 being *trans*‐regulated. Out of those 479, 131 transcripts (27%) were located on chromosome 4 (Figure 3C). Moreover, 21 genes' eQTL were mapped being co‐located with the Chorosome 4 locus, providing additional evidence that this locus is a pain sensitivity regulating locus.

### 3.4 *Rnf20* is a potential candidate with dual functioning for pain sensitivity and cognitive function

**FIGURE 4:**
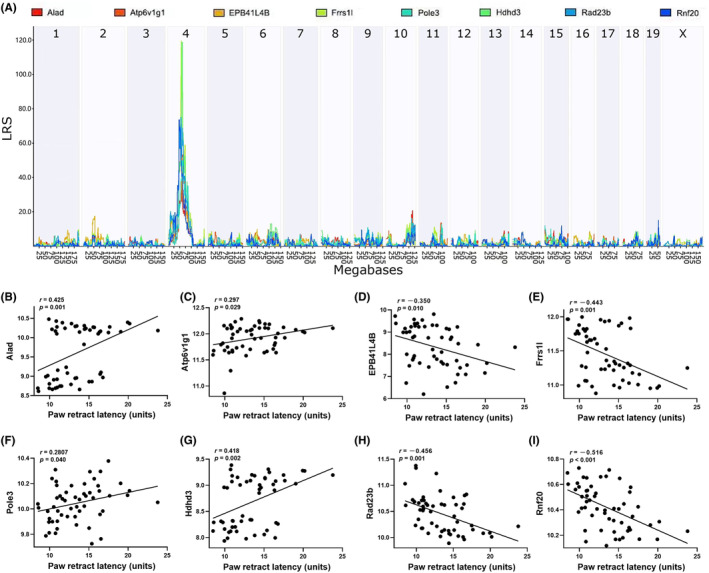
Chromosome 4 QTL candidate gene prioritization. (A) Manhattan plot showing a *cis*‐regulation of eight QTL genes (*Alad*, *Atp6v1g1*, *Epb41l4b*, *Frrs1l, Pole3*, *Hdhd3*, *Rad23b*, and *Rnf20*) at chromosome 4 QTL locus. The *X*‐axis denotes a position on the mouse genome in megabases and the *Y*‐axis indicates the LRS value. (B–I) Scatter plots showing associations between pain sensitivity (*X*‐axis, s) among BXD strains and those eight QTL genes. Each dot represents the mean value from 3 to 10 mice/strain. Pearson correlation analysis was used to determine the relationship. Pearson correlation *r* and *p* values are indicated.

Studies have shown that genetic variations largely lead to changes in levels of gene expression and thus affect the phenotype outcome.^42^ Furthermore, changes in gene expression are mostly *cis*‐regulated. Thus, identifying whether the QTL genes are regulated in *cis* by eQTL mapping provides a useful way to prioritize candidate genes. Using this approach, we identified 8 genes (*Rnf20, Rad23b, Frrs1l, Hdhd3, Alad, Epb41l4b, Atp6v1g1*, and *Pole3*) associated with pain sensitivity that have significant *cis*‐eQTLs on the chromosome 4 locus (Figure 4).

**FIGURE 5:**
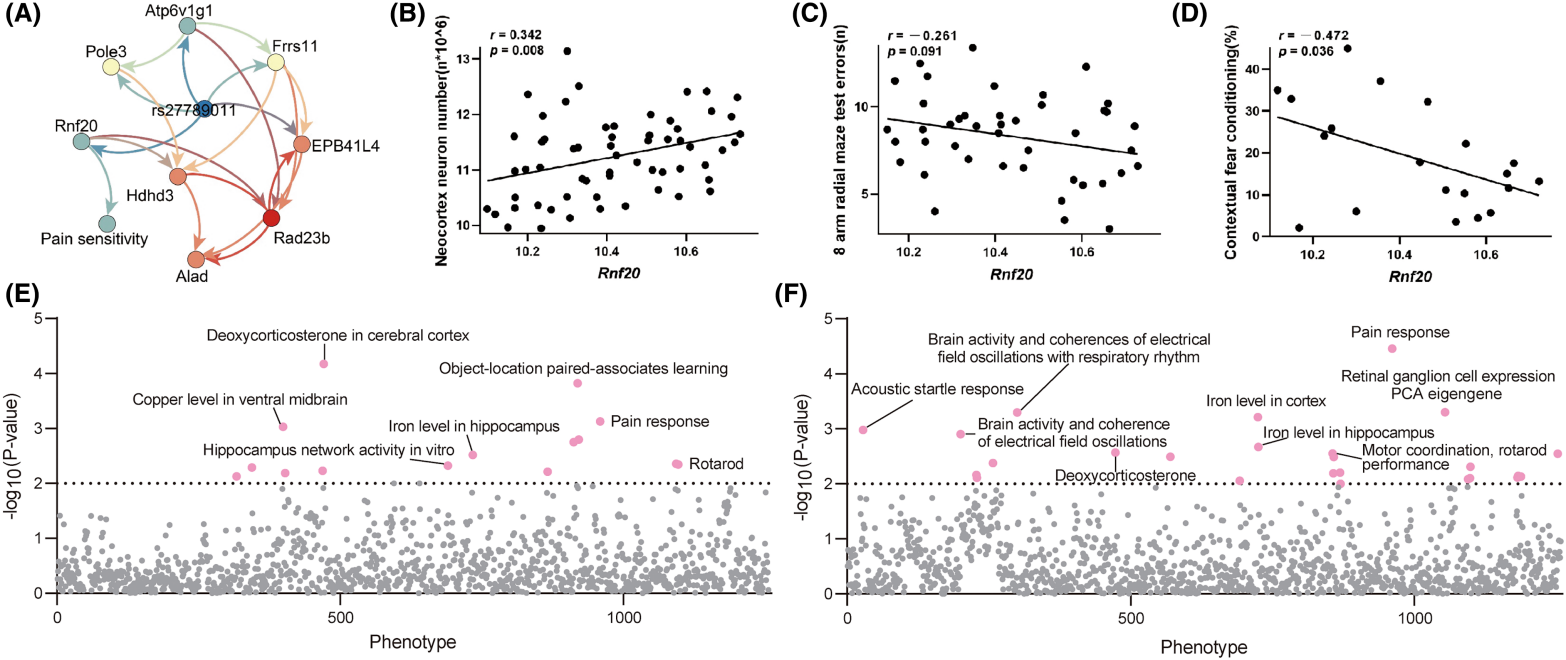
Results of joint network analysis and association studies. (A) Bayesian network structure connecting chromosome 4 QTL genotype (*rs27789011*), eight gene expression, and pain sensitivity. Edge thickness indicates edge confidence from model averaging of the 1000 highest scoring networks. (B–D) Scatter plots showing associations between *Rnf20* (*X*‐axis) and the number of neocortex neurons (B, *Y*‐axis, n*10^6), 8 arm radial maze test errors (C, *Y*‐axis, *n*), and contextual fear conditioning (D, *Y*‐axis, %). Each dot represents the mean value from 3 to 10 mice/strain. Pearson correlation analysis was used to determine the relationship. Pearson correlation *r* and *p* values are indicated in the figure. (E, F) PheWAS and ePheWAS showing the associations between *Ptn* and the central nervous system‐related traits in BXD mice. The dashed line indicates the significant threshold.

To further narrow down those genes and illustrate the potential causality with pain sensitivity, we conducted Bayesian network analysis with graphical modeling to illustrate the conditional dependencies among QTL genotype (rs27789011), 8 *cis*‐regulated genes (*Rnf20, Rad23b, Frrs1l, Hdhd3, Alad, Epb41l4b, Atp6v1g1*, and *Pole3*), and pain sensitivity. As shown in Figure 5A, we identified one pathway that links the QTL genotype and pain sensitivity through gene *Rnf20*, while other genes remained no direct connection with pain sensitivity. Taken together, our joint analysis of eQTL mapping, correlation analysis, and Bayesian network modeling suggested that *Rnf20* is the potential functional candidate for pain sensitivity on chromosome 4 QTL.

We also found strong associations (*p* < 0.05) between *Rnf20* and cognition‐related phenotypes in the BXD mice, such as Neocortex neuron number (Figure 5B), 8‐arm radial maze test errors (Figure 5C) and Contextual fear conditioning (Figure 5D). These three phenotypes were all found to be significantly correlated with the expression of *Rnf20* with Pearson coefficient *r* of 0.342, −0.261, and, −0.472, respectively. Moreover, PheWAS and ePheWAS were also revealed that *Rnf20* associated with both pain response and cognitive function‐related traits (Figure 5E,F), such as object‐location paired associates learning, motor coordination, rotoard performance, and brain activity.

### 3.5 *Rnf20* regulates pain sensitivity and cognitive function through the PI3K‐Akt signaling pathway

**FIGURE 6:**
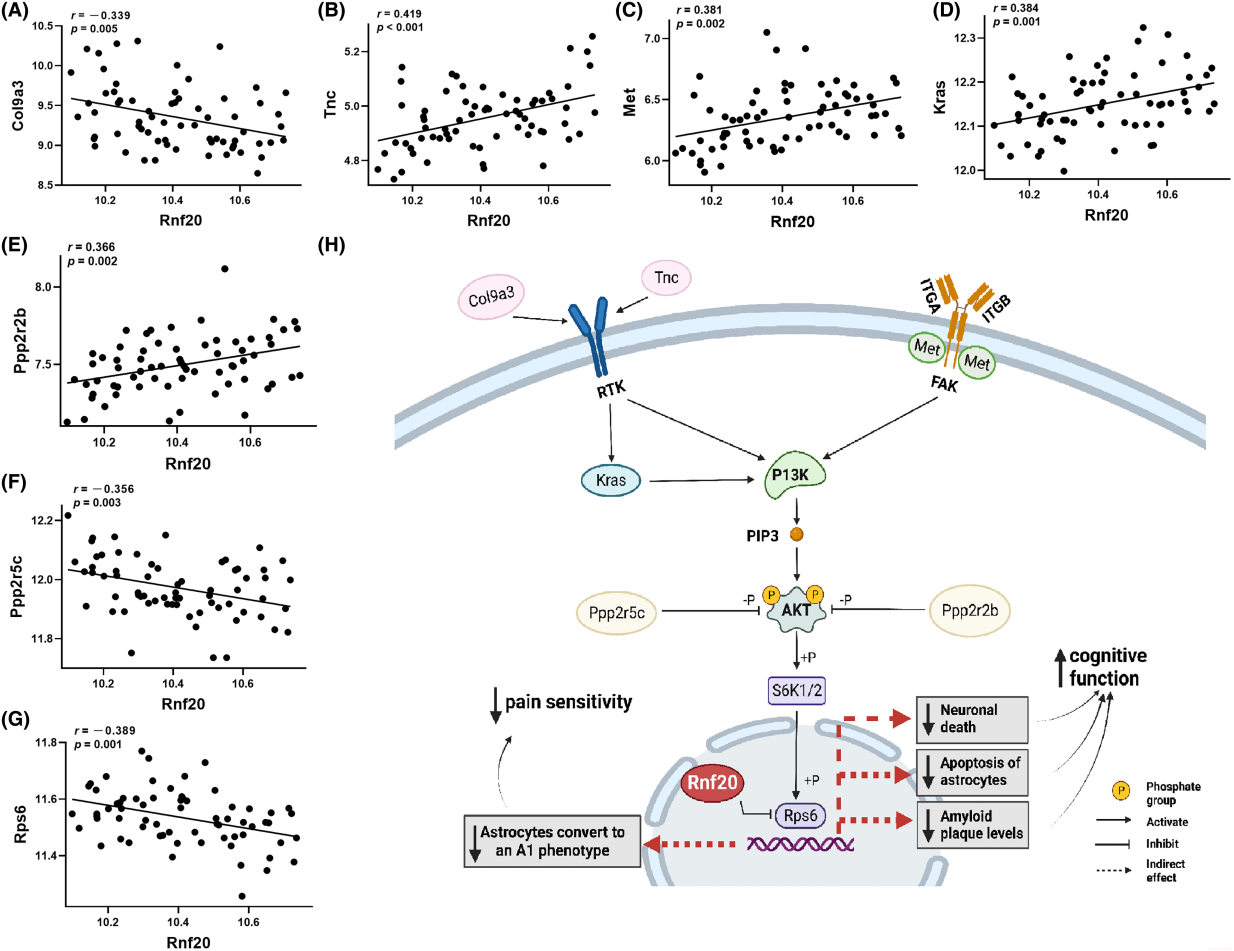
Proposed schema of *Rnf20* regulated pain sensitivity and cognitive function. (A–G) Scatter plots showing Pearson correlation associations between *Rnf20* (*Y*‐axis) and seven PI3K‐Akt signaling pathway‐related genes *Col9a3* (A), *Tnc* (B), *Met* (C), *Kras* (D), *Ppp2r2b* (E), *Ppp2r5c* (F), and *Rps6* (G). Each dot represents the mean value from 3 to 10 mice/strain. Pearson correlation *r* and *p* values are indicated in each graph. (H) Proposed schema of PI3K‐Akt signaling pathway involving these seven *Rnf20*‐associated genes. Upstream regulators of *PI3K* include *Met* (located on the cell membrane), *Col9a3* and *Tnc* (located in the extracellular matrix), and *Kras* (located in the cytoplasm downstream to *Col9a3* and *Tnc*). Activation of *PI3K* can further regulate *Akt* phosphorylation with downstream activation of *Rps6* in the nucleus through a series of signal transduction, while cytoplasmic *Ppp2r5c* and *Ppp2r2b* inhibit *Akt* activation through dephosphorylation. We propose that *Rnf20* may suppress *Rps6* to possess its dual functions in improving cognitive function by decreasing astrocyte apoptosis, neuronal death, and reducing amyloid plaque levels, and meanwhile increasing sensitivity to pain by inhibiting astrocytes converting to an A1 phenotype.

KEGG pathway analysis for the top 1000 *Rnf20* correlated genes (*p* < 0.05) identified the PI3K‐Akt signaling pathway as being significantly enriched (22 genes, *p* = 1.814e−3). Moreover, the PI3K‐Akt signaling pathway was also enriched in the pain sensitivity‐associated gene set (Figure 2A). Further intersection analysis confirmed that seven genes (*Col9a3*, *Tnc*, *Met*, *Kras*, *Ppp2r2b*, *Ppp2r5c*, and *Rps6*) in this pathway showed a correlation with both *Rnf20* and pain sensitivity (Figure 6A–G). Thus, our analysis suggested that *Rnf20* may regulate the expression of pain sensitivity and cognitive function through the PI3K‐Akt signaling pathway, particularly through interactions with the above seven genes (Figure 6H).

## 4 DISCUSSION

It is well known that individuals vary greatly in their sensitivity to pain and differences in pain sensitivity are highly heritable.^43^, ^44^, ^45^ In fact, more than 350 candidate genes have been reported to cause inter‐individual differences in pain sensitivity.^46^ For example, different strains of the BXD RI family express significant variability in pain sensitivity^13^ and *Oprd1*, *Calca*, *Mc1r*, and *TyrP1* have been shown to play an important role in the genetic variation of pain sensitivity.^47^

The neural substrates involved in pain processing and cognitive functions are interconnected. Especially, pain predominantly interferes with executive cognitive control contributing to impaired mental flexibility and attention, while cognitively demanding tasks may reduce pain perception.^48^ However, understanding of the mechanism(s) interconnected in both is still in the preliminary stage. Herein, we utilized RI strains of the BXD panel to evaluate the relationship between pain sensitivity and cognition phenotypes. To determine common genes involved in both pain sensitivity and cognition, Pearson correlation analysis between pain sensitivity phenotypes and hippocampal transcriptomic data of BXD mice was performed. It is worth noting that hippocampal genes related to pain sensitivity are directly or indirectly related to cognitive function. For instance, *Lats2*, a core component of the hippocampus signaling pathway, is considered to be highly correlated with cognitive deficits.^29^ Both human and mouse experiments support that changes in *Ptprd* expression affect cognitive function in a gene dose–response manner.^31^ Loss of *Pdpn* selectively impels long‐term synaptic inhibition in the dentate gyrus of the hippocampus, affecting learning and memory functions.^38^ In addition, the functional analysis further demonstrated that pain sensitivity‐associated genes are involved in multiple KEGG pathways implicated in both pain sensation and cognition regulation, such as the “PI3K‐Akt signaling pathway”, “MAPK signaling pathway”, “cAMP signaling pathway”, and “long‐term potentiation”.^49^, ^50^, ^51^

Our QTL mapping identified a pain sensitivity regulating locus on chromosome 4 from 46.8 to 65.9 Mb, which contains hundreds of genes. We then narrowed down those genes to 8 (*Alad*, *Atp6v1g1*, *EPB41L4B*, *Frrs1l*, *Hdhd3*, *Pole3*, *Rad23b*, and *Rnf20*) using eQTL analysis, a straightforward method to identify candidate functional genes at risk locus.^52^ Of note, Ferric Chelate reductase 1‐like encoded by *Frrs1l* is a brain‐specific protein,^53^ which is widely expressed in excitatory neurons of the cerebral cortex and hippocampus, dentate gyrus granular cells, and cerebellum Purkinje cells that play an important role in the regulation of synaptic transmission mediated by AMPA receptors.^54^ Functional loss of human *Frrs1l* can lead to severe impairment of cognitive function.^53^, ^55^ Nonetheless, our directional causality network modeling provided evidence that *Rnf20*, not *Frrs1l*, is the candidate gene involved in dual regulation of inter‐individual differences in pain sensitivity as well as cognitive functions. Ring finger protein 20 (*Rnf20*) is an E3 ubiquitin ligase that is widely involved in transcriptional regulation, DNA damage response, stem cell differentiation, and lipid metabolism.^56^, ^57^, ^58^ Highly expressed in astrocytes, *Rnf20* regulates the expression of STAT3 by mediating H2Bub1, thereby controlling the generation of neural progenitor astrocytes,^33^ which plays a crucial role in brain complexity, plasticity, and cognition. Moreover, studies have demonstrated that endothelial RNF20‐deficient mice show autistic behavior,^59^ while its overexpression in the dorsal horn induces pain hypersensitivity.^60^ Other members of the Rnf family have been similarly implicated in neurodegenerative diseases. Evidence of upregulation of *Rnf182* in the brain of AD patients suggests that *Rnf182 is* involved in the pathophysiological process of AD via induced degradation of *ATP6V0C*, a key component of the gap junction and neurotransmitter release pathway in the brain.^61^ Genetic variations in *Rnf219* increase the risk of AD,^62^ while Rnf40 has been associated with learning and memory behavior in rats.^63^

Our analysis demonstrated that *Rnf20* may affect the expression of both pain sensitivity and cognitive function through the PI3K‐Akt signaling pathway. This pathway plays a role in a variety of biological processes, such as metabolism, inflammation, cell growth, proliferation, exercise, and cancer progression,^64^, ^65^ and has also been implicated in the regulation of chronic post‐surgical pain by down‐regulating the CXCR7/PI3K/Akt signaling pathway and inducing the A1 phenotype transformation of reactive astrocytes.^66^ Furthermore, Activation of the PI3K/AKT/CREB signaling pathway in the hippocampus reduces amyloid plaque levels and improves memory decline in AD mice.^67^ Combined with the existing pathway knowledge, we mapped the regulatory network of *Rnf20* and proposed a hypothetical schema of *Rnf20* function within the PI3K‐Akt signaling pathway (Figure 6). In this, *Col9a3, Met, Tnc*, and *Kras* act as the upstream regulators of PI3K, which in turn activate AKT, while *Ppp2r5c* and *Ppp2r2b* can inhibit AKT through dephosphorylation. Activation of AKT leads to signal transduction into the nucleus, leading to the activation of the nuclear gene *Rps6*. At this point, *Rnf20*, also located in the nucleus, may influence the expression of pain and cognitive phenotypes by down‐regulating *Rps6*. Studies have shown that mycophenolate mofetil reduces neuron death and improves the cognitive impairment caused by epileptic seizure, which may be related to the decreased expression of *Rps6* in the hippocampus.^68^ Methamphetamines have been shown to down‐regulate *Rps6* expression by inhibiting the PI3K‐Akt–mTOR signaling pathway and induce astrocyte apoptosis and autophagy.^69^ We hypothesize that *Rnf20* may interfere with *Rps6*. This may reduce astrocyte apoptosis, decrease neuronal death and amyloid plaques leading to improved cognitive function, and meanwhile inhibit astrocytes converting to an A1 phenotype, thereby alleviating pain.

In summary, our study revealed potential shared biological pathways and regulatory factors between pain sensitivity and cognitive function, and ultimately identified the potential regulatory candidate gene *Rnf20*, which may be involved in the dual regulation of pain sensitivity and cognitive expression through the PI3K‐Akt signaling pathway. Although further functional analyses such as manipulation of Rnf20 and/or Rps6 using targeted treatments (e.g., antisense oligonucleotides that inhibit Rps6 in high expression strains or drugs that target these pathways) are needed to examine changes in pain sensitivity and cognition‐related phenotypes, our findings provide further insights into the molecular mechanisms involved in the crosstalk between pain sensitivity and cognitive function, and could be beneficial for prevention, clinical diagnosis and management of pain‐related neurodegenerative diseases.

## FUNDING INFORMATION

This research was funded by the Major Basic Research Project of Shandong Provincial Natural Science Foundation (ZR2019ZD27), Shandong Province Higher Educational Youth Innovation Science and Technology Program (2019KJE013), National Natural Science Foundation of China (32170989), Natural Science Foundation of Shandong Province (ZR2021MH141, ZR2023MH737), and Binzhou Medical University Research Start‐Up Fund (50012304486, 50012304309).

## CONFLICT OF INTEREST STATEMENT

The authors declare no competing interests.

## Supporting information


Table S1.

## Data Availability

Both phenotype and hippocampus transcriptome data of the BXD mice used in this study were downloaded from GeneNetwork (www.genenetwork.org). The phenotype data can be accessed with the following GN accession number: BXD_11307 for pain sensitivity, BXD_10755, BXD_10709, BXD_17942, BXD_16215, BXD_16207, and BXD_20489 for cognition related traits. All the data exhibited a normal/Gaussian distribution which was evaluated with normal probability plot function in GeneNetwork. Raw microarray data can be accessed on GEO (https://www.ncbi.nlm.nih.gov/geo/) with the identifier GSE84767. The normalized data is available on GeneNetwork under the “BXD” group and “hippocampus mRNA” type with the identifier “Hippocampus Consortium M430v2 (Jun06) RMA”.
