# Supplementary material for: Systems genetics analysis reveals the common genetic basis for pain sensitivity and cognitive function
Source: CNS Neurosci Ther. 2024 Feb 7;30(2):e14557. doi: 10.1111/cns.14557 (PMC10850811; doi:10.1111/cns.14557)
Supplement: Supplementary file 1 — Table S1. [file CNS-30-e14557-s001.docx]

**Supplementary Table 1: Lists of the top 50 pain sensitivity correlated hippocampus genes in the BXD mice.**

| **Symbol** | **Gene ID** | **Location (Chr: Mb)** | **Mean Expr** | **Max LRS** | **Max LRS Location (Chr: Mb)** | **Sample r** | **Sample p(r)** |
| --- | --- | --- | --- | --- | --- | --- | --- |
| Tfdp2 | 211586 | Chr9: 96.310545 | 6.80 | 21.2 | Chr4: 57.500003 | 0.597 | 2.31E-06 |
| Lats2 | 50523 | Chr14: 57.690214 | 8.78 | 11.1 | Chr17: 67.074057 | 0.586 | 4.06E-06 |
| C9orf150 | 52829 | Chr4: 80.953741 | 10.41 | 32.4 | Chr4: 78.900946 | 0.577 | 6.46E-06 |
| 3110009F21Rik | 67280 | Chr12: 110.151717 | 5.35 | 12.3 | Chr19: 11.096126 | -0.547 | 2.54E-05 |
| 1700051E09Rik | 67338 | Chr11: 82.858682 | 7.01 | 12 | Chr11: 59.237260 | -0.534 | 4.51E-05 |
| Fbln1 | 14114 | Chr15: 85.285845 | 8.64 | 14.8 | Chr4: 155.225671 | -0.531 | 4.92E-05 |
| Ptprd | 19266 | Chr4: 76.398275 | 6.09 | 32.9 | Chr4: 76.012861 | -0.520 | 7.75E-05 |
| Rnf20 | 109331 | Chr4: 49.656353 | 10.42 | 73.6 | Chr4: 47.699478 | -0.516 | 9.13E-05 |
| Foxn3 | 71375 | Chr12: 99.231796 | 7.61 | 8.8 | ChrX: 50.932422 | -0.515 | 9.34E-05 |
| Coasy | 71743 | Chr11: 101.086475 | 8.63 | 14.3 | Chr15: 94.266750 | -0.512 | 1.06E-04 |
| Cox10 | 70383 | Chr11: 63.963920 | 7.40 | 9.3 | Chr8: 30.622826 | -0.512 | 1.08E-04 |
| Sh3bp5l | 79566 | Chr11: 58.347169 | 10.01 | 8.7 | Chr13: 59.704246 | -0.509 | 1.20E-04 |
| Slitrk1 | 76965 | Chr14: 108.910126 | 11.83 | 12.3 | Chr7: 66.068226 | -0.508 | 1.23E-04 |
| Mylc2b | 67938 | Chr17: 70.987332 | 8.07 | 12.1 | Chr4: 13.837844 | -0.507 | 1.29E-04 |
| Sfrp1 | 20377 | Chr8: 23.447394 | 7.30 | 24.7 | Chr8: 21.960955 | 0.506 | 1.35E-04 |
| Cwf19l1 | 72502 | Chr19: 44.109447 | 7.96 | 15 | Chr16: 49.849657 | -0.500 | 1.66E-04 |
| Fam134c | 67998 | Chr11: 100.987148 | 6.01 | 10.9 | Chr4: 46.923425 | -0.500 | 1.68E-04 |
| C9orf125 | 67063 | Chr4: 49.585438 | 10.92 | 70.5 | Chr4: 47.699478 | -0.499 | 1.71E-04 |
| BM940493 | NA | Chr14: 64.586970 | 7.33 | 9.2 | Chr4: 155.894026 | -0.498 | 1.80E-04 |
| Mettl7a | 70152 | Chr15: 100.361330 | 8.06 | 15.4 | Chr13: 30.459929 | 0.497 | 1.82E-04 |
| Tbc1d9b | 76795 | Chr11: 50.171522 | 11.05 | 12.2 | ChrX: 3.231738 | -0.497 | 1.87E-04 |
| AU067697 | 100346 | Chr4: 58.962079 | 11.08 | 18 | Chr4: 58.363486 | -0.495 | 2.00E-04 |
| Tpbpb | 116913 | Chr13: 60.901456 | 5.79 | 11.2 | Chr4: 54.951001 | -0.494 | 2.03E-04 |
| Cpm | 70574 | Chr10: 117.684404 | 8.97 | 16.3 | Chr5: 137.896619 | 0.494 | 2.03E-04 |
| Pdpn | 14726 | Chr4: 143.267475 | 9.37 | 48 | Chr4: 143.189882 | 0.492 | 2.22E-04 |
| Prr24 | 66300 | Chr7: 16.272277 | 10.40 | 11.3 | Chr4: 57.500003 | -0.487 | 2.69E-04 |
| Rad23b | 19359 | Chr4: 55.390209 | 10.42 | 75 | Chr4: 54.951001 | -0.486 | 2.74E-04 |
| N28178 | 230085 | Chr4: 42.943614 | 11.65 | 84.5 | Chr4: 41.176874 | -0.484 | 2.89E-04 |
| Tspan3 | 56434 | Chr9: 56.147829 | 7.43 | 19.8 | Chr3: 57.609577 | -0.484 | 2.97E-04 |
| Erbb2ip | 59079 | Chr13: 103.898064 | 6.83 | 21.1 | Chr13: 103.151983 | 0.482 | 3.13E-04 |
| Scel | 64929 | Chr14: 103.577831 | 5.14 | 18 | Chr9: 85.118181 | -0.481 | 3.20E-04 |
| 1110007A13Rik | 210711 | Chr7: 128.714781 | 6.03 | 15.6 | Chr6: 32.610603 | -0.481 | 3.25E-04 |
| Prrg4 | 228413 | Chr2: 104.827083 | 6.47 | 13.6 | Chr5: 132.056349 | 0.477 | 3.76E-04 |
| Rbm15 | 229700 | Chr3: 107.326073 | 7.93 | 17.9 | Chr5: 137.010795 | 0.474 | 4.06E-04 |
| Akap12 | 83397 | Chr10: 4.358926 | 9.09 | 14.6 | Chr12: 10.166340 | -0.474 | 4.18E-04 |
| Tnfsf5ip1 | 107047 | Chr18: 67.647354 | 7.43 | 13.8 | Chr7: 55.607957 | -0.474 | 4.18E-04 |
| 3010001F23Rik | 75693 | ChrX: 152.416386 | 5.42 | 9.5 | Chr12: 34.927890 | -0.473 | 4.21E-04 |
| Cadm1 | 54725 | Chr9: 47.850465 | 11.23 | 15.1 | Chr12: 34.201787 | -0.473 | 4.31E-04 |
| Aida | 108909 | Chr8: 4.089356 | 10.72 | 15.4 | Chr8: 76.227149 | -0.472 | 4.34E-04 |
| 3110038A09Rik | 73126 | Chr18: 31.633710 | 6.49 | 14.2 | Chr4: 63.271283 | -0.472 | 4.37E-04 |
| Coq4 | 227683 | Chr2: 29.796765 | 9.71 | 13.7 | Chr16: 51.051146 | -0.472 | 4.42E-04 |
| AV381105 |  | Chr4: 136.358870 | 8.25 | 52.3 | Chr4: 133.964175 | -0.472 | 4.43E-04 |
| C85699 | 97104 | Chr3: 73.197882 | 6.01 | 11.4 | Chr5: 133.062783 | 0.471 | 4.55E-04 |
| Myo5a | 17918 | Chr9: 75.223099 | 11.54 | 96.7 | Chr9: 74.557367 | -0.471 | 4.58E-04 |
| E130308A19Rik | 230259 | Chr4: 59.703390 | 6.13 | 15.3 | Chr19: 58.715653 | 0.470 | 4.71E-04 |
| 9430047G12Rik | 102344 | Chr8: 36.758853 | 6.49 | 8.8 | Chr17: 21.686210 | -0.469 | 4.93E-04 |
| Tcp11 | 71841 | ChrX: 135.669409 | 6.74 | 11.3 | Chr16: 44.259473 | -0.467 | 5.19E-04 |
| Efna5 | 13640 | Chr17: 62.846058 | 7.29 | 7.9 | Chr5: 146.682242 | -0.465 | 5.59E-04 |
| Frrs1l | 100223 | Chr4: 56.960456 | 11.38 | 119.7 | Chr4: 57.274213 | -0.463 | 5.84E-04 |
| 0610007P08Rik | 76251 | Chr13: 63.898847 | 6.19 | 9.7 | Chr10: 102.470556 | -0.463 | 5.91E-04 |
